# Potential of Cheese-Associated Lactic Acid Bacteria to Metabolize Citrate and Produce Organic Acids and Acetoin

**DOI:** 10.3390/metabo13111134

**Published:** 2023-11-06

**Authors:** Luana Faria Silva, Tássila Nakata Sunakozawa, Diego Alves Monteiro, Tiago Casella, Ana Carolina Conti, Svetoslav Dimitrov Todorov, Ana Lúcia Barretto Penna

**Affiliations:** 1 Institute of Biosciences, Humanities and Exact Sciences, Food Engineering and Technology Department, UNESP—São Paulo State University, São José do Rio Preto 15054-000, SP, Brazil; luanafarianutricionista@gmail.com (L.F.S.); tassilanakata@gmail.com (T.N.S.); diego8monteiro@gmail.com (D.A.M.); ac.conti@unesp.br (A.C.C.); 2 Department of Dermatological, Infectious and Parasitic Diseases, FAMERP—São José do Rio Preto Medical School, São José do Rio Preto 15090-000, SP, Brazil; tiago.casella@famerp.br; 3 ProBacLab, Department of Food Science and Experimental Nutrition, Faculty of Pharmaceutical Sciences, USP—São Paulo University, São Paulo 05508-000, SP, Brazil; slavi310570@abv.bg; 4 CISAS—Center for Research and Development in Agrifood Systems and Sustainability, Escola Superior de Tecnologia e Gestão, Instituto Politécnico de Viana do Castelo, 4900-347 Viana do Castelo, Portugal

**Keywords:** citrate metabolism, proteolysis, technological application, flavoring compounds, biopreservation

## Abstract

Lactic acid bacteria (LAB) are pivotal in shaping the technological, sensory, and safety aspects of dairy products. The evaluation of proteolytic activity, citrate utilization, milk pH reduction, and the production of organic compounds, acetoin, and diacetyl by cheese associated LAB strains was carried out, followed by Principal Component Analysis (PCA). Citrate utilization was observed in all *Leuconostoc* (*Le*.) *mesenteroides, Le. citreum, Lactococcus* (*Lc*.) *lactis, Lc. garvieae,* and *Limosilactobacillus* (*Lm*.) *fermentum* strains, and in some *Lacticaseibacillus* (*Lact*.) *casei* strains. Most strains exhibited proteolytic activity, reduced pH, and generated organic compounds. Multivariate PCA revealed *Le. mesenteroides* as a prolific producer of acetic, lactic, formic, and pyruvic acids and acetoin at 30 °C. *Enterococcus* sp. was distinguished from *Lact. casei* based on acetic, formic, and pyruvic acid production, while *Lact. casei* primarily produced lactic acid at 37 °C. At 42 °C, *Lactobacillus* (*L*.) *helveticus* and some *L. delbrueckii* subsp. *bulgaricus* strains excelled in acetoin production, whereas *L. delbrueckii* subsp. *bulgaricus* and *Streptococcus* (*S.*) *thermophilus* strains primarily produced lactic acid. *Lm. fermentum* stood out with its production of acetic, formic, and pyruvic acids. Overall, cheese-associated LAB strains exhibited diverse metabolic capabilities which contribute to desirable aroma, flavor, and safety of dairy products.

## 1. Introduction

Lactic acid bacteria (LAB) constitute a microbial group naturally present in the autochthonous microbiota of various types of food. They are also applied in the food, pharmaceutical, and chemical industries due to their technological and health promoting properties. Several LAB are involved in numerous food fermentation processes, with most of them being described as “Generally Recognized As Safe” (GRAS) by the United States Food and Drug Administration (FDA). They play an essential role in the production of various commercial products [1,2]. LAB strains isolated from different ecological environments can be associated with different metabolic properties, and their long history of application in the preparation of various fermented food products is a scientific fact [3].

LAB are divided into two groups based on their ability to metabolize glucose and the type of produced metabolites. The homofermentative metabolic group possesses aldolase, an enzyme associated with the conversion of glucose almost quantitatively to lactic acid via glycolysis or the Embden–Meyerhof–Parnas pathway. Examples of LAB in this group include *Lactococcus*, *Pediococcus*, *Enterococcus*, *Streptococcus*, and some lactobacilli species [4].

Heterofermentative LAB, on the other hand, produce the enzyme phosphoketolase and ferment glucose to produce lactic and acetic acids, ethanol, and carbon dioxide (CO_2_) via the phosphoketolase pathway. Typical examples of heterofermentative LAB include representatives from *Leuconostoc*, *Oenococcus*, *Weissella*, and some lactobacilli [5,6].

When lactose, the main carbohydrate in milk, is metabolized by LAB, it can lead to the production of various organic acids, both volatile and nonvolatile, as well as aromatic compounds. These compounds play a crucial role in determining the quality of fermented food products. Furthermore, specific organic acids found in fermented dairy products can originate from various sources, including the hydrolysis of milk fat (resulting in free fatty acids such as acetic or butyric acids) by LAB, production or technological application as acidulants (such as citric and lactic acids), biochemical metabolism (involving citric, orotic, and uric acids), and/or bacterial growth (leading to lactic, acetic, pyruvic, propionic, and formic acids) [6].

Furthermore, LAB can metabolize several other carbohydrate sources, including citrate, as an alternative carbon source. The metabolism of citric acid in LAB depends on the permease citrate enzyme (CitP) and can serve as the sole energy source for LAB or be co-metabolized [7]. In the case of heterofermentative LAB, such as *Le. mesenteroides* and *Lm. fermentum*, citrate is converted into pyruvate, which is subsequently reduced to lactate. The presence of citrate leads to the generation of additional energy during sugar degradation [8,9]. Specifically, in the case of *Lact. casei* (a facultative heterofermentative lactobacilli), some strains can metabolize citrate when the concentration of glucose is limited. However, in homofermentative LAB, such as *Lc. lactis* and *Enterococcus* spp., pyruvate serves as the common intermediate compound formed during both carbohydrate and citrate metabolism. The subsequent conversion of pyruvate results in the production of C4 aroma compounds, including acetaldehyde, acetoin, 2,3-butanediol, and diacetyl [10]. Furthermore, the metabolism of lactose and citrate by LAB can also yield significant quantities of ethanol, acetic acid, and other flavor-related molecules that are highly valued in fermented dairy products [8,11].

Regardless of the carbohydrate metabolism pathways (homo and heterofermentative) used by LAB, the final metabolites from the fermentation processes are primarily characterized by the accumulation of organic acids and a decrease in the pH values [3,12]. In addition to contributing to the sensory characteristics of dairy products, the production of various organic acids is essential for product preservation due to the reduction in pH. Furthermore, low pH values play a crucial role in casein precipitation during the production of fermented milk products and in specific properties required for soft cheese production, enabling easy plasticization and stretching of the curd in hot water [13]. Low pH has a bactericidal and/or bacteriostatic effect against some pathogenic microorganisms, contributing to an extension of shelf life and enhanced food safety [14,15,16].

Nowadays, several studies evaluating the production of flavor compounds and organic acids by LAB have been conducted, followed by multivariate analyses of produced compounds [17,18]. Nevertheless, the direct addition of LAB to food matrices, such as milk, is more interesting because it allows for their real potential application. In this context, this study aimed to characterize the indigenous LAB strains present in buffalo Mozzarella cheese. We assessed their proteolytic activity through the production of extracellular proteases in agar milk, their ability to utilize citrate in a differential medium, their capacity to reduce milk pH, and their ability to produce organic acids, acetoin, and diacetyl in skim milk.

## 2. Materials and Methods

### 2.1. LAB Strains

In this study, sixty-seven LAB strains of different biotypes from various genera, including *Le*. *mesenteroides*, *Le*. *citreum*, *Enterococcus* spp., *E*. *durans*, *E*. *faecium*, *Lact. casei*, *L. delbrueckii* subsp. *bulgaricus*, *L. helveticus*, *Lc. lactis*, *Lc. garvieae, Lm. fermentum*, and *S. thermophilus* were used. These strains were previously isolated from Brazilian buffalo Mozzarella cheese [19]. All strains belong to the São Paulo State University (UNESP) Culture Collection (CCLAB-UNESP, WDCM 1182), Brazil, and were maintained as stock cultures at −80 °C in De Man, Rogosa and Sharpe (MRS) (Difco Laboratories, Detroit, MI, USA) or M17 broth (Himedia, Mumbai, MH, India) supplemented with 20% glycerol (*w*/*v*; Sigma-Aldrich, Saint Louis, MO, USA).

### 2.2. Growth Evaluation of the LAB Strains

In pursuit of a standardized exponential cell phase inoculum (10^6^–10^8^ CFU/mL), preliminary experiments were conducted to determine the viable counts of each species. Two or more strains representing each genus/species were cultured at different temperatures and incubation times to ensure inoculation with a consistent number of viable cells. For each experiment, overnight cultures were employed. The optimal incubation conditions for each species in the specific media (agar, broth, and milk) are summarized in Table 1.

The cultures were reactivated from frozen stocks in MRS or M17 broth, followed by streaking onto agar medium and incubated under specific conditions (as detailed in Table 1). To determine pH values and organic acids using High Performance Liquid Chromatography (HPLC) (see below), one colony was collected from each agar plate using a sterile loop and cultured in 6 mL of MRS and/or M17 broth at 30 °C or 37 °C for 18 h (as indicated Table 1). This growth procedure was repeated once, and the analyses were performed in duplicate.

### 2.3. Citrate Utilization by the LAB Strains

The citrate utilization by the LAB strains was assessed in duplicate using the differential medium proposed by Kempler and McKay [20]. In this medium, citrate-positive colonies were described as blue, while citrate-negative colonies appeared white after 48 h of incubation at appropriate temperatures and under anaerobic conditions generated by Anaerobac (Probac, São Paulo, Brazil). *Lc. lactis* subsp. *lactis* biovar. *diacetylactis* ATCC 11007 was used as a positive control.

### 2.4. LAB Strains Proteolytic Activity

The proteolytic activity of LAB strains was qualitatively evaluated on skim milk agar plates following the method outlined by Pailin et al. [21]. The medium was composed of 50 g/L peptone (Sigma, Saint Louis, MO, USA ), 30 g/L yeast extract (Sigma), 12 g/L agar, and 10% (*v*/*v*) reconstituted skim milk (Difco). The strains from agar plate were inoculated using a sterile loop (approximately 10 µL) and then incubated at either 30 or 37 °C for mesophilic bacteria (depending on the species) and at 42 °C for thermophilic bacteria, for 24 h, in duplicate (as specified in Table 1). The presence of clearing zones, indicative of proteolysis, was recorded.

### 2.5. Inoculum Preparation and Fermentation

The strains were initially grown with a 2% starting inoculum in 6 mL of M17 or MRS broth, as previously described in Table 1. Cells were collected by centrifugation (5000× *g* for 6 min at 4 °C), washed and resuspended using a sterile saline solution (2%, *w/v*). Each strain was inoculated individually (2%, *v/v*) into reconstituted skim milk (RSM, Molico, Nestlé, Araçatuba, SP, Brazil) at 10% (*w/v*), prepared with sterile distilled water, to achieve a target concentration of approximately 10^6^ CFU/mL. After inoculation, samples were incubated at 30 °C, 37 °C or 42 °C for 6 h and 18 h (as indicated Table 1) and evaluated for the changes in pH and the production of organic acid, acetoin, and diacetyl, all in duplicates.

#### 2.5.1. Determination of pH Values

At 6 h and 18 h of fermentation times, aliquots from the fermented milk samples were collected, and the pH was measured using a pH meter (model PG1800, Gehaka, São Paulo, SP, Brazil), in duplicate. Control samples (non-inoculated sterile RSM) were also incubated and analyzed under the same conditions.

#### 2.5.2. Analysis of Organic Acids, Acetoin, and Diacetyl

The analyses of organic acids, acetoin, and diacetyl production were conducted using HPLC, as described by Donkor et al. [22], in duplicate. For this analysis, 3.0 mL of fermented milk samples were withdrawn at 6 h and 18 h of fermentation and then mixed with 80 μL of 15.5 M nitric acid. Non-inoculated sterile RSM was assessed and quantified as a control. Subsequently, the samples were diluted with 1.0 mL of the 0.01 M sulfuric acid (the mobile phase used in the HPLC analysis). The resulting mixture was centrifuged (15,000× *g* for 20 min at 4 °C) using an Eppendorf 5415R centrifuge (Eppendorff, Hamburg, Germany) to remove proteins. The supernatant was then filtered through 0.20 μm membrane filters (Millipore, Billerica, MA, USA), collected into HPLC vials, and analyzed using a Flexar™ LC system (PerkinElmer, Waltham, MA, USA), and the separation was achieved using an Aminex HPX-87H column (Bio-Rad Laboratories, Hercules, CA, USA). The column was stabilized for at least 3 h with the mobile phase under conditions identical to those of the chromatographic experimental procedure. Concentrations of the organic acids were estimated based on standard curves obtained using solutions of pre-determined concentrations [22,23]. Unidentified peaks were not reported.

#### 2.5.3. Principal Component Analysis

Principal component analysis (PCA) was conducted to characterize studied LAB strains in terms of their production of organic acids and acetoin, and pH alteration. Given the potential application of LAB in various food fermentation processes, this analysis was performed separately for mesophilic (30 °C or 37 °C) and thermophilic (42 °C) LAB, as well as for different fermentation times. Prior to analysis, the data of organic acids, acetoin, and pH were fixed in columns (variables) and the strains in rows (cases). The PCA was carried out using the correlation matrix and without factor rotation. Data analysis was performed using the statistical package Statistica 7.0 (StatSoft Inc., Tulsa, OK, USA).

## 3. Results

### 3.1. LAB Growth, Citrate Fermentation, and Production of Extracellular Protease

Faster bacterial growth within the first 12 h of incubation was observed for *Enterococcus* spp., *S. thermophilus*, and *L. helveticus* cultures, while for other cultures, optimal growth occurred between 12 h and 18 h. An important preliminary step influencing all subsequent results was the adjustment of the optimal concentration of viable bacterial cells. Generally, two successive revitalizations in broth under optimal growth conditions for each species yielded better results for most LAB strains (as detailed in Table 1). Additionally, all strains of *Le. mesenteroides*, *Le. citreum, Lc. lactis, Lc. garvieae*, and *Lm. fermentum*, and the majority of *Lact. casei* (except the *Lact. casei* SJRP66) exhibited a positive-citrate profile. The studied cultures were also capable of producing extracellular protease in milk agar (except for *Le. mesenteroides* SJRP160), as evidenced by the formation of a hydrolysis halo around the inoculated strains.

### 3.2. Reduction in pH and Production of Organic Compounds

**Figure 1:**
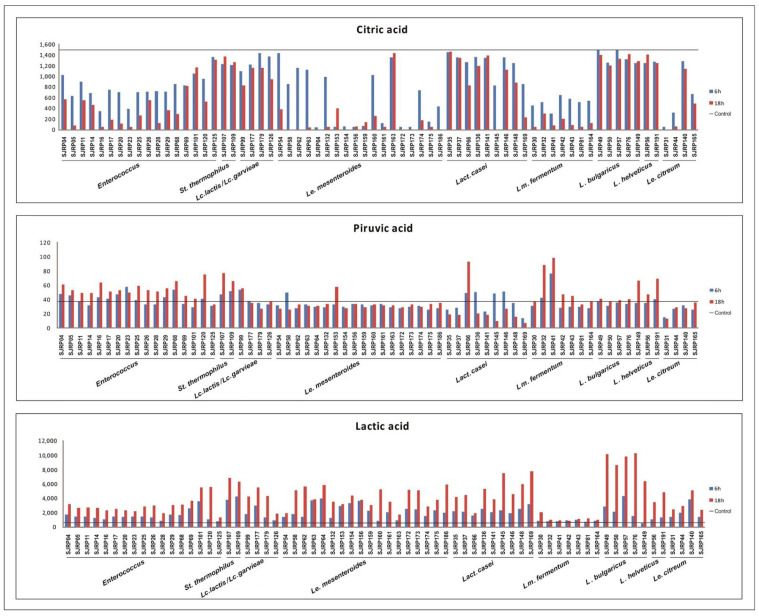
Concentration (mg/L) of citric, lactic, and pyruvic acids produced by LAB strains. The strains were incubated in optimum temperature for 6 h and 18 h. Control—milk before fermentation.

**Figure 2:**
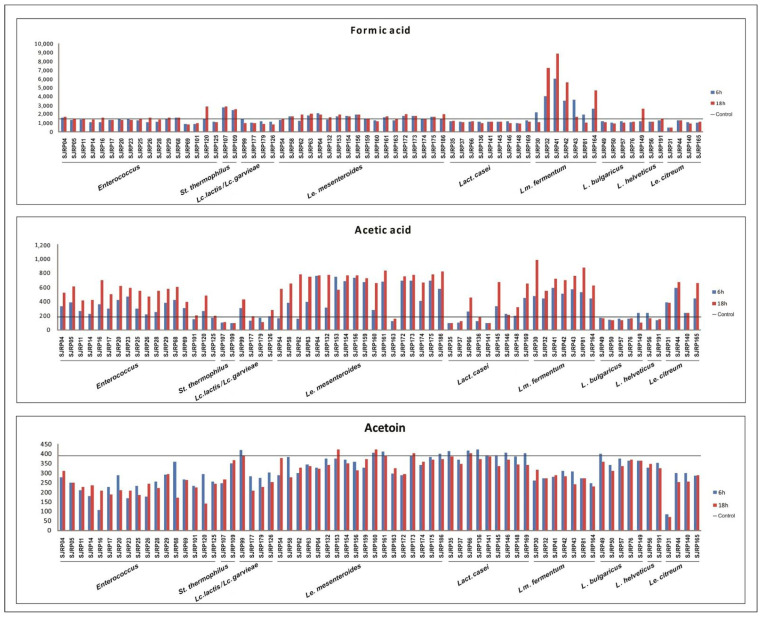
Concentration (mg/L) of formic and acetic acids and acetoin produced by LAB strains. The strains were incubated in optimum temperature for 6 h and 18 h. Control—milk before fermentation.

The production of fermented dairy products relies on the selection of LAB starter cultures with specific acidification properties. Changes in pH and production of metabolites by LAB play a vital role in determining the technological properties of starter cultures, including organoleptic characteristics and the safety of the final products. The ability of LAB to produce acetoin and organic acid at 6 h and 18 h of the fermentation process is illustrated in Figure 1 and Figure 2 and their contribution to milk pH reduction (from an initial 6.2 ± 0.3) is shown in the Supplementary Material (Table S1).

The following compounds were identified and quantified: citric acid (1494 mg/L), pyruvic acid (37 mg/L), lactic acid (596 mg/L), formic acid (1418 mg/L), acetic acid (193 mg/L), and acetoin (395 mg/L). These compounds were also detected in non-inoculated sterile RSM (control) samples after 18 h. It is worth noting that the applied analytical procedures were not efficient in quantifying other organic acids often present in fermented dairy, such as orotic acid, uric acid, or diacetyl, which may require further exploration in further studies.

In general, most of the strains were able to reduce the milk pH value to 5.0, as observed at 18 h of fermentation. Notably, *Enterococcus* spp. SJRP101 and *S. thermophilus* SJRP107 and SJRP109 demonstrated a rapid reduction of the milk pH to 5.0 compared with the other tested strains. Throughout the fermentation of RSM, pH values progressively decreased due to the production of organic acids. During the monitored 18 h fermentation period, only 50% of *Le. mesenteroides* strains were able to reduce the pH value of milk to ≤5.0. Similarly, to the tested *Le. mesenteroides* strains, the investigated *Le. citreum* strains demonstrated limited acidifying potential. Only *Le. citreum* SJRP31 managed to reduce the milk pH to <5.0 by the end of the fermentation period (18 h) and did not produce high concentrations of acids. This study highlights the species-specific nature of organic compound production by LAB.

Most species of *Enterococcus* strains (57%), including *Enterococcus* spp., *E. durans*, and *E. faecium* were able to reduce the milk pH to ≤5 at 18 h of fermentation. Overall, this resulted in higher production of lactic acid and lower pH values. For example, *Enterococcus* sp. SJRP101 and *Enterococcus* sp. SJRP125, when fermenting RSM, exhibited the highest production of lactic acid (Figure 1) and the lowest pH values after the fermentation period. Additionally, all tested *Enterococcus* spp. strains produced pyruvic and acetic acid, while formic acid production was observed only in *Enterococcus* sp. SJRP04, SJRP16, and SJRP120. Notably, acetoin production was not observed in any of the tested *Enterococcus* spp., *E. durans*, and *E. faecium* strains when compared with the control (Figure 2).

Most of the investigated *Lact. casei* strains (90%) were able to reduce the milk pH to ≤5 at 18 h of the fermentation process. Citrate assimilation by these microorganisms varied, with some showing complete, partial, or no assimilation of citric acid (Figure 1). Among these strains, *Lact. casei* SJRP145 and SJRP169 produced the highest concentrations of lactic and acetic acids, resulting in the lowest amounts of citric and pyruvic acid and the lowest pH values (4.42 and 4.36, respectively). In contrast, milk fermented by *Lact. casei* SJRP66 exhibited higher pH values (5.52) and greater production of pyruvic acid and acetoin at 18 h of fermentation, probably associated with citrate metabolism (Figure 1 and Figure 2). Notably, the production of formic acid was not detected in these cultures.

All the investigated *Lc. lactis* strains successfully reduced the milk pH to ≤5.0 during the fermentation period. Among them, *Lc. lactis* SJRP177 exhibited the lowest pH (4.38) during fermentation and the highest concentration of lactic acid. Furthermore, *Lc. lactis* SJRP177 showed partial consumption of citric acid. On the other hand, *Lc. lactis* SJRP99 demonstrated the highest consumption of citric acid, along with the highest concentrations of pyruvic and acetic acids, as well as acetoin. Notably, the production of formic acid was not detected in any of the investigated *Lc. lactis* strains.

In this study, the two tested *S. thermophilus* strains demonstrated faster milk acidification compared with other strains, with a pH of 4.8 and 4.7 for SJRP107 and SJRP109, respectively, at 6 h of fermentation. These strains also produced substantial amounts of lactic and formic acids. Although low concentrations of pyruvic acid (≤77 mg/L) were detected, the tested *S. thermophilus* strains did not produce other organic acids. Interestingly, all compounds exhibited a reduction in comparison with the initial concentration in the milk sample (control) (Figure 1 and Figure 2).

All tested cultures of *L. bulgaricus* successfully reduced the milk pH to ≤5 at 18 h of fermentation, except for *L. bulgaricus* SJRP149. Furthermore, for most *L. bulgaricus* strains, a significant reduction in pH during the last 12 h of fermentation was observed. *L. bulgaricus* produced the highest concentration of lactic acid (11863 mg/L) at 18 h of fermentation (Figure 1). However, the production of citric acid, formic acid, acetic acid, and acetoin was not detected in this species. None of *Lm. fermentum* strains were able to acidify milk to a pH ≤ 5 at 18 h of fermentation. However, the production of organic acids was detected in all *Lm. fermentum* strains, while acetoin was not observed. Generally, *Lm. fermentum* exhibited slow fermentation of milk and produced low amounts of lactic acid (≤2078 mg/L) (Figure 1). In contrast, *Lm. fermentum* strains produced the highest concentrations of pyruvic and formic acids (99 mg/L and 8863 mg/L, respectively, for *Lm. fermentum* SJRP41) and acetic acid (986 mg/L for *Lm. fermentum* SJRP30) (Figure 1 and Figure 2). In this study, *L. helveticus* acidified milk to a pH ≤ 5 by the end of fermentation (18 h). Additionally, the tested *L. helveticus* strains produced pyruvic and lactic acid, although the production of acetoin and other organic acids was not detected.

### 3.3. Principal Component Analysis (PCA)

**Figure 3:**
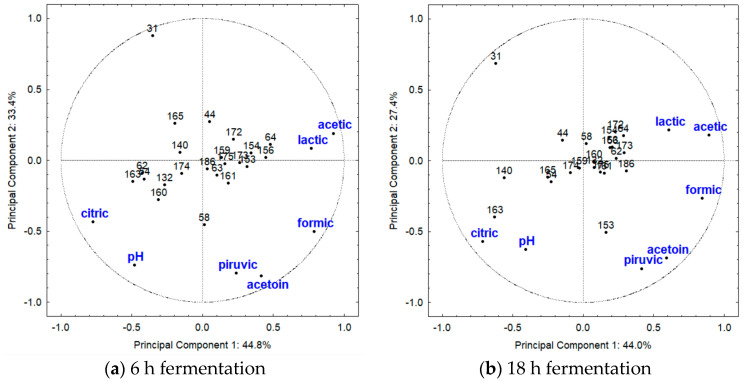
Results of first and second principal components evidenced by Principal Component Analysis based on the production of organic acid and acetoin, and pH values of milk fermented by mesophilic LAB (30 °C). At 6 h of fermentation (**a**), component 1 was primarily influenced by citric, lactic, formic, and acetic acids, while component 2 was associated with pyruvic acid, acetoin, and pH values. At 18 h of fermentation (**b**), similar patterns were observed, except for lactic acid and pH. The strains are coded by numbers; see Table 1 for complete notation of strains.

To better evidence the relationships among the LAB strains and the organic acids and acetoin, a PCA was carried out using the relative amounts of these compounds for all strains reported in Table 1. For mesophilic LAB (Table 1) cultivated at 30 °C, the first and second principal components (PC) explained 44.8% and 33.4% of observed variations, respectively, totaling 78.2% at 6 h of fermentation (Figure 3a). Component 1 was primarily influenced by citric, lactic, formic, and acetic acids, while component 2 was associated with pyruvic, acetoin, and pH values. At 18 h of fermentation (Figure 3b), the first and second components explained 44.0% and 27.4% of observed variations, totaling 71.4%. Similar patterns were observed, except for lactic acid and pH, in comparison with the results at 6 h. Additionally, at 6 h of fermentation, the highest concentration of pyruvic acid (50 mg/L, Figure 1) distinguished *Le. mesenteroides* SJRP58 from the other LAB strains. At 18 h of fermentation, two *Le. mesenteroides* strains could be clearly differentiated using PCA. *Le. mesenteroides* SJRP153 exhibited the highest production of acetoin and pyruvic acid, while *Le. mesenteroides* SJRP163 showed the lowest consumption of citric acid and the highest pH value. The genetic diversity among *Le. mesenteroides* strains, as previously reported by Silva et al. [19], could explain these variations in physiological behavior. Furthermore, *Le. citreum* SJRP31 stood out from all other LAB at both fermentation times (Figure 3). This strain completely consumed all the citric acid and did not produce any of the studied compounds (Figure 1 and Figure 2).

*Leuconostoc* sp., especially *Le. mesenteroides* subsp. *cremoris*, have the capability to produce significant amounts of diacetyl and other C4 compounds, such as acetoin, from citrate present in milk [24,25,26]. Interestingly, some strains of *Le. citreum*, like the SJRP31 strain (Figure 1), exhibited high consumption of citric acid, even though there was no apparent relationship with the production of any of the analyzed compounds. In this case, it is possible that other unexamined compounds were being produced.

**Figure 4:**
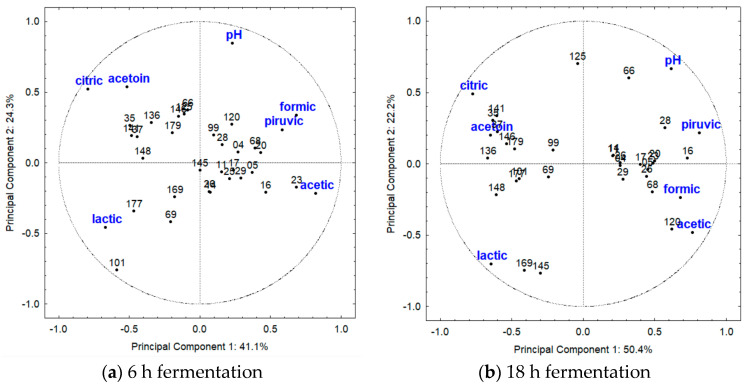
Results of first and second principal components evidenced by Principal Component Analysis based on the production of organic acid and acetoin, and pH values of milk fermented by mesophilic LAB (37 °C). At 6 h of fermentation (**a**), component 1 was influenced by citric and acetic acids, while component 2 was associated with pH value. At 18 of fermentation (**b**), component 1 was influenced by citric, pyruvic, and acetic acids, while component 2 was associated with lactic acid value. The strains are coded by numbers; see Table 1 for complete notation of strains.

For the mesophilic LAB cultivated at 37 °C (Table 1), at 6 h of fermentation, the PCA revealed that the first and the second PC described 41.1% and 24.3% of the variability, respectively, totaling 65.4% (Figure 4). At 18 h of fermentation, the PC explained 50.4% (component 1) and 22.2% (component 2) of the variance, totalizing 72.6% of observed variation (Figure 4). At 6 h of fermentation (Figure 4a), citric and acetic acids contributed to explaining the variance of component 1, while pH value explained the variance of component 2. *Enterococcus* spp. strains were mainly characterized by the highest concentration of acetic acid (467 mg/L; Figure 1), particularly strain SJRP23, likely due to the metabolism of citric acid. In contrast, most strains of *Lact. casei* were distinguished by the highest concentration of citric acid, especially *Lact. casei* SJRP35 (1456 mg/L; Figure 1). Notably, *Enterococcus* sp. SJRP23 was genetically distinct from the other *Enterococcus* spp. strains, as reported in a previous study [19].

At 18 h of fermentation (Figure 4b), citric, pyruvic, and acetic acids, which were strongly associated with *Enterococcus* spp. strains, contributed to explaining the variance of component 1, while for component 2, lactic acid explained the variations. *Lact. casei* SJRP145 and *Lact. casei* SJRP169 differed from other LAB by the lactic acid production (7511 mg/L and 7661 mg/L, respectively; Figure 1), and the amount of this acid was significantly higher compared with the other strains. On the other hand, *Enterococcus* sp. SJRP120 was characterized by the production of acetic acid. These variations among *Enterococcus* spp. strains can likely be attributed to genetic polymorphism, as there is a high genetic diversity among the strains, which were grouped into distinct clusters at the 85% similarity level [19].

**Figure 5:**
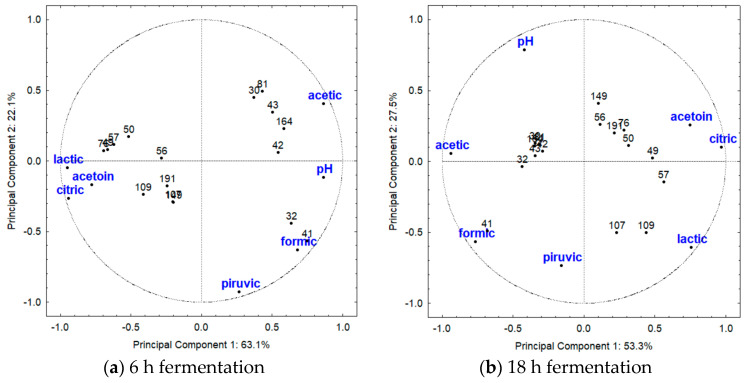
Results of first and second principal components evidenced by Principal Component Analysis based on the production of organic acid and acetoin and pH values of milk fermented by thermophilic LAB (42 °C). At 6 h of fermentation (**a**), component 1 was influenced by citric, lactic, formic, and acetic acids and acetoin, while component 2 was associated with pyruvic acid and pH value. At 18 of fermentation (**b**), component 1 was influenced by citric, lactic, and acetic acids, pH value, and acetoin, while component 2 was associated pyruvic acid. The strains are coded by numbers; see Table 1 for complete notation of strains.

For the thermophilic LAB (Table 1), at 6 h of fermentation, the PCA showed that the PC described 80.8% of the variability, with 53.3% attributed to the first component and 27.5% to the second components. Meanwhile, at 18 h of fermentation, the PC explained 63.1% (component 1) and 22.1% (component 2), totalizing 85.2% of observed variation (Figure 5). At 6 h of fermentation (Figure 5a), citric, lactic, formic, and acetic acids, as well as acetoin, contributed to explaining the variability of component 1, while pyruvic acid and pH value explained the variance of component 2. *L. bulgaricus* and *L. helveticus* strains were grouped due to their production of acetoin and citric acid, as they produced higher amounts of acetoin compared with the other LAB, and in this species, citric acid was neither produced nor metabolized (Figure 1 and Figure 2). Acetoin was likely produced as a result of lactose metabolism. *S. thermophilus* SJRP107 and SJRP109, as well as *L. bulgaricus* SJRP57, stood out from the other LAB due to their highest production of lactic acid (Figure 1). Homofermentative lactobacilli and *S. thermophilus* utilize the glycolytic pathway to produce energy and convert at least 85% of lactose into lactic acid.

All strains of *Lm. fermentum* were grouped together and distinguished from the other thermophilic LAB by their high production of acetic acid (Figure 2). However, *Lm. fermentum* SJRP41 stood out among all LAB due to its exceptional production of formic acid (6038 mg/L; Figure 2).

At 18 h of fermentation (Figure 5b), citric, lactic, and acetic acids, along with pH value and acetoin, explained the variability of component 1, while pyruvic acid contributed to explain the variance of component 2. *Lm. fermentum* SJRP32 and SJRP41 were grouped together and distinguished from the other *Lm. fermentum* strains by their production of acetic acid and pH value. These strains belong to the same cluster based on the banding pattern polymorphism at a similarity level of 85% [19]. These strains produced higher amounts of acetic acid compared to the other LAB, especially *Lm. fermentum* SJRP30 (986 mg/L; Figure 2), likely due to the citric acid metabolism. Furthermore, even though pyruvic acid could not characterize the *Lm. fermentum* strains, this species produced the highest amounts of pyruvic acid (Figure 1). *L. bulgaricus* strains, in contrast to other thermophilic LAB, were characterized by their production of lactic and citric acids, along with acetoin. They produced the highest levels of lactic acid (Figure 1).

## 4. Discussion

LAB produce a rich array of metabolites which are critical in shaping the unique technological and sensory profile of each fermented food variety.

### 4.1. LAB Growth, Citrate Fermentation, and Production of Extracellular Protease

The investigated *Enterococcus* spp., *S. thermophilus*, and *L. helveticus* strains exhibited faster growth during the initial 12 h of fermentation compared with the other LAB strains. Thermophilic LAB, such as *S. thermophilus* and *L. helveticus*, as well as *Enterococcus* spp., have the ability to rapidly convert lactose into lactic acid through the glycolytic pathway. These LAB species play essential roles in the traditional production of long-ripened Italian cheeses like Parmesan and Swiss-type cheeses such as Emmental and Gruyere [27,28]. *E. faecalis, E. faecium,* and *E. durans* are among the most common microorganisms in Mediterranean-type cheese. They make significant contributions to proteolysis, lipolysis, amino acid degradation, and citrate fermentation [29].

Although the use of citrate by *Enterococcus* spp. was not observed in this study using a differential medium, the consumption of citric acid was detected through HPLC analysis, showing a significant reduction in most experiments. The glycolysis and citrate metabolism in some *Enterococcus* species provide them with an additional energetic advantage during their growth [30]. These metabolic processes result in the formation of acetate, acetaldehyde, diacetyl, acetoin, and 2,3-butanediol from pyruvate. This is particularly important for flavor formation during milk fermentation and the subsequent ripening of fermented dairy products [31].

In this study, all strains of *Le. mesenteroides*, *Le. citreum*, *Lc. lactis*, *Lc. garvieae*, and *Lm. fermentum*, and most of *Lact. casei* (except for *Lact. casei* SJRP66), exhibited a positive-citrate utilization profile. The ability to metabolize citric acid by LAB depends on the permease citrate (CitP) enzyme [7], which is present in various genera of LAB, including *Leuconostoc* and lactobacilli [9], and *Enterococcus* [19]. *Leuconostoc* species are heterofermentative LAB and can produce CO_2_ and volatile compounds besides organic acids, which contribute to the flavor and texture of fermented cream [14].

Citrate fermentation plays a direct role in the flavor and quality of dairy products, and its outcome varies depending on the strain used. There are two distinct genetic configurations in LAB, each involving a variety of enzymes and transporters. In both pathways, the initial step involves the action of citrate lyase, which catalyzes the cleavage of internalized citrate into oxaloacetate (OAA) and acetate. Following the initial step, two diverging pathways are observed in LAB, leading to the production of either succinate or pyruvate. The pathway leading to succinate, via malate and fumarate, is commonly found in many mesophilic non-starter LAB species. In the second pathway, OAA is further decarboxylated to pyruvate and CO_2_ by OAA decarboxylase (CitM), thereby contributing to the central pool of pyruvate in the glycolytic pathway for carbohydrate and citrate co-metabolism [32,33].

The ability to metabolize citrate is of paramount importance when selecting appropriate cultures for food fermentation processes. This metabolism can result in the production of various compounds, including acetic acid, acetate, formate, ethanol, acetaldehyde, acetoin, 2,3-butanediol, diacetyl, and CO_2_ [34]. Some of these compounds significantly contribute to the development of aroma and flavor development in fermented foods, particularly in fermented dairy products. Additionally, citrate metabolism plays a role in the production of CO_2_, which is responsible for the formation of characteristic “eyes” in certain types of cheese [26,35]. Moreover, citrate metabolism has been linked to the production of aroma compounds from amino acids, with the transamination of amino acids by aminotransferases serving as a crucial step in the formation of aromatic compounds by LAB [36].

All tested LAB strains, except for *Le. mesenteroides* SJRP160 demonstrated the production of extracellular protease when cultured on milk agar. Although lactobacilli are not typically known for being prolific producers of extracellular proteases, certain LAB, such as *L*. *bulgaricus*, *Lact*. *casei*, *Lact. paracasei, Lact. rhamnosus*, *Lactiplantibacillus plantarum*, and *L. helveticus* possess the capability to hydrolyze proteins through their proteolytic system. This is achieved by the action of cell envelope proteinases, specifically serine proteinases [37]. This attribute is a highly relevant characteristic of LAB, with significant potential for application in the production fermented food [38].

In dairy product manufacturing, the proteases produced by both starters and non-starters LAB play a crucial role in hydrolysis of milk proteins. This process releases essential amino acids that support microbial growth and, as a result, significantly impact the specific texture, flavor, and aroma characteristics of the final fermented products. Therefore, when seeking LAB strains for use in food fermentation processes where the production of flavor compounds and the development of desirable textures are important objectives, it is imperative to consider the strains’ capacity to metabolize citric acid and their proteolytic activity. These factors are key in selecting the most suitable candidates for such applications.

### 4.2. Reduction in pH and Production of Organic Compounds and Acetoin

The investigated LAB demonstrated the ability to reduce milk pH, to produce organic acid and acetoin during the fermentation process (Figure 1 and Figure 2). In addition to their contribution to technological and sensory properties, organic acids produced by LAB have the potential to prevent spoilage and enhance food taste, thereby improving consumer acceptance and appeal.

The quantities of organic compounds (citric, pyruvic, lactic, formic and acetic acids and acetoin) produced by LAB cultures were significant, particularly the highest concentration of lactic acid produced by *S. thermophilus* and *L. bulgaricus*, as well as the highest levels of formic and acetic acid produced by most *Lm. fermentum* strains. Generally, in all fermentation setups, the production of lactic acid increased between 6 and 18 h. However, establishing a standard behavior for the other tested organic acids and acetoin was not possible, as they exhibit strain-specific characteristics. In such a case, it is worth considering the assimilation or conversion of these compounds into others, although these were not assessed in this study (Figure 1 and Figure 2). The organic acids identified in this study are commonly produced by LAB, and are also found in soft cheeses and fermented milk products [39].

*Le. mesenteroides* exhibited a low acidification profile during the monitored 18 h fermentation period, a common characteristic of this species. *Le. mesenteroides* is generally associated with slow milk acidification [40]. However, it finds extensive use in the dairy industry for the production of aromatic compounds and exopolysaccharides as adjunct cultures [41]. As expected for these LAB, high or total consumption of citric acid (compared to the control) at 18 h of fermentation was detected by HPLC analysis, except for *Le. mesenteroides* SJRP163. Additionally, a high production of acetic acid and acetoin was observed (825 and 422 mg/L, respectively) (Figure 1 and Figure 2). Lower pH values during fermentation by *Le. mesenteroides* SJRP64, combined with the highest consumption of citric acid and the highest concentration of lactic, formic, and acetic acids at 6 h of fermentation (4000 mg/L, 2105 mg/L, and 760 mg/L, respectively) were observed (Figure 2). Furthermore, some strains of *Le. mesenteroides* can produce antimicrobial substances against pathogenic and spoilage bacteria. In a previous study, it was observed that *Le. mesenteroides* subsp. *mesenteroides* SJRP55 caused a reduction in *L. monocytogenes* population and had a distinguished effect on fatty acid profile; it increased conjugated linoleic acid and decreased α-linolenic and oleic acid contents [14].

*Le. citreum* strains produced a low concentration of acids, consistent with the known low acid tolerance of this species [42]. The highest concentration levels of acetic and lactic acids were produced by *Le. citreum* SJRP44 and SJRP140, respectively, which are organic acids commonly produced by this species [43]. However, the production of other monitored compounds was not detected in these strains (Figure 1 and Figure 2). Furthermore, lactic, and acetic acids are important antifungal substances produced by *Le. citreum* [44], which is also desirable for food production industries.

*Enterococcus* spp. is considered a good producer of acids through lactose fermentation, and some strains are also capable of metabolizing citric acid or citrate to produce various aromatic compounds [29]. While the production of acetoin in milk by enterococci has been reported [29,45], this ability was not observed in any of the tested *Enterococcus* spp. strains. *Enterococcus* spp. can also exhibit a crucial technological role in several fermented food products because of their specific metabolite characteristics and their ability to withstand heat stress and other adverse environmental conditions. They are present in the microbiota of different fermented products, such as dairy, meat, fish, seafood, and vegetables. Moreover, in addition to organic acids, some strains of *Enterococcus* can produce antimicrobial substances, short-chain fatty acids, and volatile compounds that can improve the safety and sensorial properties of the fermented products [29,45].

The *Lact. casei* strains demonstrated the ability to produce organic acids and to assimilate citrate. These microorganisms are commonly used in mixed or pure cultures to produce fermented dairy products such as milk or cheese, which require slow fermentation processes. It is likely that *Lact. casei* strains utilize pyruvate and/or citrate metabolism to produce high concentration of lactic and acetic acids [46]. Citrate metabolism could potentially result in the production of pyruvate, acetate, and acetoin [8,47].

During the fermentation period, the production lactic and acetic by the tested *Lc. lactis* strains was observed. According to Maślak et al. [48], during fermentation, *Lc. lactis* converts lactose directly into lactic acid, thereby increasing the rate of lactic acid formation. Due to the high production of pyruvic and acetic acids, as well as acetoin, *Lc. lactis* SJRP99 appears to be a promising candidate for producing important aromatic compounds in dairy products. In milk, *Lc. lactis* can co-metabolize carbohydrates and citrate as secondary carbon energetic sources, leading to the generation of CO_2_ and C4 aroma compounds, which can enhance the organoleptic characteristics of dairy products [49]. However, it is important to note that only specific variants of *Lc. lactis*, such as *Lc. lactis* subsp. *lactis* biovar *diacetylactis*, possess the ability to utilize citrate. This property is linked to the presence of a plasmid-encoded citrate transporter gene [49,50]. Additionally, there is a valid hypothesis suggesting the acquisition of the citrate-fermenting capacity by some lactococci, possibly through horizontal transfer of plasmid genes [51].

Both strains of *S. thermophilus* acidified milk faster than other LAB strains during the fermentation process. These *S. thermophilus* strains have been previously evaluated for their technological properties [52]. It is a type of LAB often used in cheese, yogurt, and other types of fermented dairy production due to its fast metabolism. When present, it is mainly responsible for the production of lactic acid [53]. Additionally, this species also produces formic and pyruvic acids. In the processing of fermented products such as yogurt, there exists a symbiotic relationship between *L. bulgaricus* and *S. thermophilus.* The formic and pyruvic acids produced by *S. thermophilus* are important for the growth of *L. bulgaricus*. The formic acid produced by *S. thermophilus* is utilized by *L. bulgaricus*, and in turn, *L. bulgaricus* supports the growth of *S. thermophilus* through the production of peptides or amino acids [54].

The investigated *L. bulgaricus* strains produced organic acids and reduced the pH value during the fermentation period, except for *L. bulgaricus* SJRP149. This difference is likely related to a smaller count of viable cells in the inoculum (approximately 10^6^ CFU/mL) compared with the other strains of this species. Generally, *L. bulgaricus* thrives at acidic pH levels and is often responsible for post-acidification in fermented milks products [55].

In this study, *Lm. fermentum* strains were unable to acidify milk (pH ≤ 5) during the fermentation period. However, they produced the highest concentrations of pyruvic, formic, and acetic acids compared with the other tested LAB strains, as demonstrated by Hashemi et al. [56]. According to Ayad et al. [57], *Lm. fermentum* ferments dairy products slowly, likely due to the production of low amounts of lactic acid. The production of acetic acid is probably influenced by the citric acid metabolism, as previously reported by other LAB strains, and as observed for these microorganisms [8].

Although *L. helveticus* is not typically known as a good producer of organic acids, the tested strains were able to acidify milk to pH ≤ 5 during the fermentation process. However, *L. helveticus* strains with lower acidifying ability can still find utility as adjunct cultures in dairy products, depending on their other essential properties. For instance, their efficient proteolytic system is responsible for producing enzymes that impart distinctive characteristics in terms of texture, flavor, and aroma to various products [58,59,60].

The production of organic acids by any starter culture can also help distinguish the beneficial and pathogenic properties of LAB. Additionally, the specificity of the nutritional matrix can influence the metabolic properties of the microorganisms, and the formation of metabolites can be a result of secondary reactions rather than direct involvement in the principal pathways of the microorganisms [61]. The exchange of genetic material, including plasmid DNA, among microorganisms (horizontal and vertical gene transfer) can lead to atypical fermentation characteristics in the evaluated strains and introduce additional beneficial characteristics or pathogenic properties. For example, this can involve the ability to metabolize different carbohydrates [62] or to inherit antibiotic resistance or virulence factor properties [63].

Another important point to be considered is the performance of LAB in pasteurized and unpasteurized milk. The use of raw milk in cheese production offers unique sensory properties and enhances cheese quality. These sensory characteristics are directly linked to various milk enzymes, particularly the physiological and biochemical properties of indigenous LAB found in raw milk or the dairy environment. These microorganisms play a key role in producing a significant amount of lactic acid through lactose breakdown, leading to rapid milk acidification. The LAB also contributes to the production of various compounds that influence cheese flavor and texture, thus enhancing the overall cheese product. Raw milk’s natural microbiota adds complexity and depth to the cheese, making it a preferred choice for those seeking distinctive and high-quality cheese varieties [15].

### 4.3. Principal Component Analysis (PCA)

The PCA was applied to the relative amounts of organic acids and acetoin to demonstrate the relationships among the mesophilic LAB cultivated at 30 °C, mesophilic LAB cultivated at 37 °C, and thermophilic LAB strains (Figure 4 and Figure 5). Genetic diversity among mesophilic LAB strains could explain variations in their physiological behavior. Some thermophilic LAB strains were distinguished from the other LAB by their highest production of lactic acid. These microorganisms utilize the glycolytic pathway to assimilate carbohydrates. In this case, pyruvate serves as a key intermediate compound in their metabolism and can be converted into various end products, including lactic, formic, and acetic acids, acetaldehyde, ethanol, acetoin, diacetyl, and butane-2,3-diol [53,54].

*Lm. fermentum* strains were distinguished from all LAB by the highest production of formic and acetic acids. The high concentrations of these acids can contribute to the inhibition of *Listeria* sp. in dairy products [6]. These strains can also be used to control other pathogenic microorganisms in dairy industries. However, it is important to note that high amounts of acetic acid can result in vinegar-flavored dairy, which may be unappealing [64]. In another study, the capacity of synthesizing a significant concentration of acetic acid by *Lm. fermentum* was also observed [65], along with a high concentration of pyruvate, which can serve as an antioxidant [66]. Moreover, some strains, like *Lm. fermentum* SJRP30, can present good survivability under simulated gastrointestinal tract conditions, and are revealed to be safe and to possess similar or superior probiotic characteristics compared to the reference strain *L. rhamnosus* GG (ATCC 53103) [67].

This study also revealed significant differences in the concentration of organic compounds produced by LAB strains. The relationship between the LAB species and the production of the organic acids and acetoin during fermentation at different temperatures was better evidenced by PCA.

Currently, many studies have focused on the bioactive metabolites produced by LAB, such as short chain-fat acids, exopolysaccharides and antimicrobial compounds which can contribute to the safety, stability, flavor, and aroma of food products [8]. Some LAB strains can beneficially modulate the host’s metabolism; those are known as probiotics [14,29]. They are live microorganisms that, when administered in adequate amounts, confer a health benefit to the host [68]. However, it is essential to consider the difficulty of reproducing the microorganism’s behavior in the food matrix compared with those cultivated in synthetic medium, since the generation of bioactive metabolites can vary with incubation, substrates, and other processing conditions.

It is important to mention that the selection of LAB for application in fermented products is a critical process, primarily influenced by several key factors. Firstly, the specific product’s desired sensory attributes, such as flavor, texture, and aroma, play a pivotal role in choosing the right strains. Secondly, the food matrix (animal or vegetable origin) in which the fermentation will take place, including temperature, pH, and available nutrients, must be considered to ensure the selected bacteria can grow. LAB use their biochemical machinery to assimilate the nutrients from the matrix to produce important metabolites (organic acids, polyols, and exopolysaccharides), and thus have a great number of applications in the food industry. Additionally, some LAB strains can also increase the nutritional value and safety of food products by the production of vitamins and antimicrobial substances. Ultimately, the safety and stability of the selected LAB strains are crucial, as certain strains may produce undesirable byproducts or pose health risks. Then, the information provided by this study can aid in selecting the best species and/or strains to produce various fermented foods. Furthermore, this study highlights the potential of novel indigenous LAB strains to metabolize citrate and to produce high concentrations of organic acids and acetoin.

In essence, the successful application of LAB in fermented products hinges on a thoughtful evaluation of these multifaceted factors to achieve the desired product quality and safety.

## 5. Conclusions

The ability to metabolize citrate in a differential medium was observed for all *Le. mesenteroides*, *Le. citreum*, *Lc. lactis,* and *Lm. fermentum,* and some strains of *Lact. casei*. Additionally, except for *Le. mesenteroides* SJRP160, the other strains produced extracellular proteases. Most LAB strains acidified milk to a pH ≤ 5.0 during 18 h of fermentation and produced organic acids and acetoin. This production is species- or strain-dependent. Furthermore, the relationship between LAB species and the production of organic acids and acetoin during fermentations was better evidenced through PCA. *Le. mesenteroides* strains were characterized by their production of pyruvic acid and acetoin, whereas *Enterococcus* spp. strains exhibited the ability to produce acetic, formic, and pyruvic acids. *Lact. casei* strains, on the other hand, were characterized by their production of lactic acid. *L. helveticus* and certain *L. delbrueckii* subsp. *bulgaricus* strains were characterized by their ability to produce acetoin, while the production of lactic acid was associated with all *L. delbrueckii* subsp. *bulgaricus* and *S. thermophilus* strains. The production of acetic, formic, and pyruvic acids was a feature of *Lm. fermentum*.

Finally, this study suggests the potential of cheese-associated LAB strains that can impart interesting aroma and flavor to the fermented product while also contributing to product safety. The different LAB strain profiles observed in this study encourage us to perform further investigations using multi-omics and data integration analyses for better understanding the relationships between LAB strains and metabolites, to estimate the production of other important compounds to increase LAB applicability and to enhance the safety and quality of fermented dairy products.

## Figures and Tables

**Table 1:** Indigenous lactic acid bacteria strains isolated from buffalo Mozzarella cheese, and conditions of revitalization and growth in agar, broth and milk.

Characteristic	Species	Strains	Broth/Agar *		Milk	
			Medium/O_2_	T °C/48 h	O_2_	T °C/18 h
Mesophilic	*Le. mesenteroides*	SJRP54, SJRP58, SJRP62, SJRP63, SJRP64, SJRP132, SJRP153, SJRP154, SJRP156, SJRP159, SJRP160, SJRP161, SJRP163, SJRP172, SJRP173, SJRP174, SJRP175, SJRP186	MRS/AE	30	AE	30
	*Le. citreum*	SJRP31, SJRP44, SJRP140, SJRP165	MRS/AE	30	AE	30
	*Enterococcus* spp.	SJRP04, SJRP11, SJRP16, SJRP23, SJRP69, SJRP101, SJRP120, SJRP125	MRS/AE	37	AE	37
	*E. durans*	SJRP05, SJRP14, SJRP17, SJRP25, SJRP26, SJRP29, SJRP68	MRS/AE	37	AE	37
	*E. faecium*	SJRP20, SJRP28	MRS/AE	37	AE	37
	*Lact. casei*	SJRP35, SJRP37, SJRP66, SJRP136, SJRP141, SJRP145, SJRP146, SJRP148, SJRP169	MRS/AN	37	AE	37
	*Lc. lactis* and *Lc. garvieae*	SJRP99, SJRP177, SJRP179, SJRP126	M17/AE	37	AE	37
Thermophilic	*S. thermophilus*	SJRP107, SJRP109	M17/AE	42	AE	42
	*L. bulgaricus*	SJRP49, SJRP50, SJRP57, SJRP76, SJRP149	MRS/AN	42	AE	42
	*L. helveticus*	SJRP56, SJRP191	MRS/AN	42	AE	42
	*Lm. fermentum*	SJRP30, SJRP32, SJRP41, SJRP42, SJRP43, SJRP81, SJRP164	MRS/AN	42	AE	42

* MRS—De Man, Rogosa and Sharpe; AE—aerobic atmosphere; AN—anaerobic atmosphere.

## Data Availability

All data generated by the current project are available upon request. Data is not publicly available due to privacy.

## Supplementary Materials

The following supporting information can be downloaded at: https://www.mdpi.com/article/10.3390/metabo13111134/s1, Table S1: Determination of pH values at 6 h and 18 h of fermentation.
