# Supplementary material for: Potential of Cheese-Associated Lactic Acid Bacteria to Metabolize Citrate and Produce Organic Acids and Acetoin
Source: Metabolites. 2023 Nov 6;13(11):1134. doi: 10.3390/metabo13111134 (PMC10673126; doi:10.3390/metabo13111134)
Supplement: Supplementary file 1 [file metabolites-13-01134-s001.zip › metabolites-2685506-supplementary.pdf]

metabolites

MDPI

Article

Potential of Cheese-Associated Lactic Acid Bacteria to Metabolize Citrate and Produce Organic Acids and Acetoin

Luana Faria Silva [1], Tássila Nakata Sunakozawa [1], Diego Alves Monteiro [1], Tiago Casella [2], Ana Carolina Conti [1], Svetoslav Dimitrov Todorov [3,4] and Ana Lúcia Barretto Penna [1,*]

[1] Institute of Biosciences, Humanities and Exact Sciences, Food Engineering and Technology Department, UNESP-São Paulo State University, São José do Rio Preto 15054-000, SP, Brazil; luanafarianutricionista@gmail.com (L.F.S.); tassilanakata@gmail.com (T.N.S.); diego8monteiro@gmail.com (D.A.M.); ac.conti@unesp.br (A.C.C.); ana.lb.penna@unesp.br (A.L.B.P)
[2] Department of Dermatological, Infectious and Parasitic Diseases, FAMERP-São José do Rio Preto Medical School, São José do Rio Preto 15090-000, SP, Brazil; tiago.casella@famerp.br
[3] ProBacLab, Department of Food Science and Experimental Nutrition, Faculty of Pharmaceutical Sciences, USP–São Paulo University, São Paulo 05508-000, SP, Brazil; slavi310570@abv.bg
[4] CISAS-Center for Research and Development in Agrifood Systems and Sustainability, Escola Superior de Tecnologia e Gestão, Instituto Politécnico de Viana do Castelo, 4900-347 Viana do Castelo, Portugal
* Correspondence author: ana.lb.penna@unesp.br; Tel. +55-17-3221-2266

Supplementary Material

Table S1: Determination of pH values at 6 h and 18 h of fermentation.

Characteristics	Species	Strains	pH	
			6h	18h
Mesophilic	*Le. mesenteroides*	SJRP54	5.9	5.5
		SJRP58	5.8	5.4
		SJRP62	6.0	5.3
		SJRP63	5.7	4.8
		SJRP64	5.6	4.8
		SJRP132	5.9	5.3
		SJRP153	5.8	5.1
		SJRP154	5.7	4.8
		SJRP156	5.6	4.8
		SJRP159	5.8	5.3
		SJRP160	6.0	4.9
		SJRP161	5.8	5.3
		SJRP163	6.0	5.5
		SJRP172	5.7	4.8
		SJRP173	5.7	4.8
		SJRP174	5.9	5.4
		SJRP175	5.9	5.3
		SJRP186	5.8	5.3
	Le. citreum	SJRP31	5.5	4.8
		SJRP44	5.6	5.2
		SJRP140	5.7	5.5

		SJRP165	5.6	5.4
	Enterococcus	SJRP04	5.5	4.9
		SJRP05	5.5	5.1
		SJRP11	5.5	4.9
		SJRP14	5.5	5.0
		SJRP16	5.7	5.5
		SJRP17	5.6	5.1
		SJRP20	5.7	5.2
		SJRP23	5.5	5.2
		SJRP25	5.5	5.0
		SJRP26	5.5	5.0
		SJRP28	5.9	5.7
		SJRP29	5.4	4.8
		SJRP68	5.4	4.8
		SJRP69	5.3	4.4
		SJRP101	4.7	4.4
		SJRP120	5.8	4.8
		SJRP125	5.9	5.6
	Lact. casei	SJRP35	5.7	4.9
		SJRP37	5.7	4.8
		SJRP66	5.8	5,6
		SJRP136	5.7	4.0
		SJRP141	5.7	4.9
		SJRP145	5.5	4.4
		SJRP146	5.7	4.8
		SJRP148	5.6	4.5
		SJRP169	5.5	4.4
	Lc. garvieae	SJRP126	5.8	5.5
	Lc. lactis	SJRP99	5.5	4.8
		SJRP177	5.1	4.4
		SJRP179	5.7	4.6
Thermophilic	*S. thermophilus*	SJRP107	4.8	4.4
		SJRP109	4.7	4.3
	L. bulgaricus	SJRP49	5.3	3.8
		SJRP50	5.4	3.8
		SJRP57	5.1	3.8
		SJRP76	5.6	5.9
		SJRP149	6.0	3.8
	L. helveticus	SJRP56	5.7	4.8
		SJRP191	5.7	4.6
	Lm. fermentum	SJRP30	5.6	5.3

	SJRP32	5.7	5.6
	SJRP41	5.5	5.5
	SJRP42	5.5	5.5
	SJRP43	5.4	5.3
	SJRP81	5.4	5.2
	SJRP164	5.5	5.4
